# Prevalence and influencing risk factors of eczema among preschool children in Urumqi city: a cross-sectional survey

**DOI:** 10.1186/s12887-021-02819-5

**Published:** 2021-08-16

**Authors:** Haonan Shi, Guangsheng Wan, Tingting Wang, Jia Zhu, Lan Jiang, Shaowei Ma, Jian Yao, Zhe Yin, Murizhati Maimaiti, Huijuan Dong

**Affiliations:** 1 School of Public Health, Xinjiang Medical University, Urumqi, 830011 China; 2 School of Nursing & Health Management, Shanghai University of Medicine & Health Sciences, No.279, Zhouzhu Highway, Pudong New District, Shanghai, 201318 China; 3 Cadre Health Center, People’s Hospital of Xinjiang Uygur Autonomous Region, Urumqi, 830001 China; 4 Department of Laboratory Medicine, Xinjiang Uyghur Autonomous Region Maternal and Child Health Hospital, Urumqi, 830002 China

**Keywords:** Indoor environment, Preschool children, Eczema, Pets

## Abstract

**Background:**

Eczema is a chronic inflammatory disease associated with impaired quality of life. We identified indoor environmental risk factors, to provide strong evidence for the prevention and control of eczema in preschool children.

**Methods:**

Using a cross-sectional study with stratified random cluster sampling, we conducted a self-administered questionnaire survey among 8153 parents of children aged 2–8 years in 60 kindergartens in six districts of Urumqi city during August 2019.

**Results:**

Among 8153 preschool children, 12.0% of the children have been diagnosed with eczema. Multivariate logistic regression analysis showed that caesarean section (odds ratio [OR] = 1.18, 95% confidence interval [CI]: 1.03–1.36), being an only child (OR = 1.36, 95% CI: 1.18–1.57), presence of mould or moisture in the mother’s home before pregnancy (OR = 1.53, 95% CI: 1.17–2.00), presence of flies or mosquitoes in the dwelling currently (OR = 1.31, 95% CI: 1.10–1.55), pets kept in the child’s home currently (OR = 1.23, 95% CI: 1.01–1.51), presence of pets during child’s first year (OR = 1.45, 95% CI: 1.14–1.85), and family history of eczema (OR = 3.53, 95% CI: 2.98–4.19) are the risk factors for the development of eczema, whereas ethnicity other than the Han Chinese (OR = 0.77, 95% CI: 0.61–0.96) is a protective factor for eczema.

**Conclusion:**

Preschool children in Urumqi are at a high risk of eczema, particularly those of the Han Chinese ethnicity. Parents should be attentive to the indoor living environment of children and take actions to reduce indoor humidity, pest control and elimination, and avoid raising pets to reduce the risk of development of eczema in children.

**Supplementary Information:**

The online version contains supplementary material available at 10.1186/s12887-021-02819-5.

## Background

Eczema is an allergic skin condition with symptoms including skin damage, swelling, itchiness and recurrent rashes. The symptoms in children have a complex aetiology, usually believed as combination of environmental and genetic factors [1]. Eczema causes itchiness, which can lead to sleep disturbances in children and even affect physical and mental development in severe cases. Eczema can progress into asthma or rhinitis; moreover, the treatment of eczema and its triggering factors can impose an economic burden on the patient [2–4].

Many domestic and international studies have analysed the factors associated with the prevalence of eczema in preschool children. A study conducted in 10 provinces and cities in China, which was a survey analysis of acute allergies and eczema, showed that the prevalence of eczema in children ranged from 4.8 to 15.8% [5]. In contrast, two studies in the United States and Sweden showed that the prevalence of eczema was 20 and 23%, respectively [6, 7]. In recent years, China’s economy has developed rapidly, the demand for housing has increased, and a large number of families are keeping pets at home. Eczema has been associated with animal contact, poor ventilation, and living in large urban areas having heavy traffic [8]. Located in the central part of Xinjiang, Urumqi is a multi-ethnic city with a hot and dry climate. Moreover, due to the specific lifestyle of the people in Xinjiang, the incidence of asthma, pneumonia, and allergic rhinitis is relatively high [5, 9]; however, large-scale epidemiological investigation of eczema has not been conducted. There is a lack of research on the prevalence of eczema and the related influencing factors in minority population areas of Xinjiang, and considerably less research on the risk factors of eczema in preschool children.

Therefore, considering the actual situation and specific environmental conditions in Urumqi, this survey was conducted in August 2019 in 60 kindergartens in Urumqi’s six districts: Xinshi, Saybag, Tian Shan, Shuimugou, Toutunhe, and Midong. We interviewed parents of 8153 preschool children regarding their indoor living conditions and the prevalence of eczema. The risk factors influencing the prevalence of eczema in preschool children were investigated with the intention to identify relevant indoor environmental factors that can affect childhood eczema. The results of this survey are summarized and analysed to provide recommendations for future prevention and control of eczema in preschool children in Xinjiang.

## Methods

### Subjects

In this cross-sectional study, children were selected using a stratified random cluster sampling method from daycare centres in Urumqi. Stratified by six administrative areas in Urumqi (including Xinshi, Tian Shan, Toutunhe, Saybag, Midong, and Shuimogou), eight to twelve daycare centres in each administrative area were randomly selected. The parents of all children in each daycare centre were invited to participate in the questionnaire survey. The parents of 8153 children in 60 day-care centres received the questionnaire. The ethnic distribution of responders was predominantly Han, with fewer ethnic minority participants, mainly composed of Uyghurs, Kazaks and other unnamed minorities, accounting for approximately 13.1%. The survey was conducted in August 2019.

### Questionnaire

The questionnaire survey [5] was conducted in day-care centres, this was a 4-stage process as shown in Table S1. We contacted the Urumqi Education Bureau and the head of each kindergarten and conducted unified professional training for the teachers in charge of particular classes before the survey. The questionnaires were sent to the kindergartens, and the teachers distributed them to the parents, who were allowed to take the questionnaire home and were supposed to complete and return it within 1 week to the teacher in charge of the class. The teachers of all classes then sent the questionnaire to the head of the kindergarten. The head of the school collected all completed questionnaires from the kindergarten and sent them to the Urumqi Education Bureau.

The questionnaire was prepared in accordance with the ALLHOME-2 in Naydenov’s [10] doctoral thesis and the questionnaire used by Bornehag in the Dampness in Buildings and Health survey study [11], with minor revisions based on specific conditions in China and Urumqi. The survey had the following six components: (1) demographic characteristics, including sex, ethnicity, education level and home address; (2) feeding status, including whether the child is an only child, whether the child was breastfed, breastfeeding duration, and the age at which the child attended kindergarten; (3) health conditions of the child and family members, including wheezing, asthma, pneumonia, allergic rhinitis and other related symptoms; (4) living environment for the child, including the type of housing, whether it is furnished, and the presence of new furniture, ventilation, and smoke extraction; (5) living habits, including animals, plants, frequency of cleaning, and smoking habit of the child’s family members; and (6) eating habits, including type and frequency of food consumption. The completed questionnaires were investigated by two or more trained members of the team and incomplete questionnaires were eliminated.

Eczema was considered to be present if parents reported a clear diagnosis. The frequency of cleaning the child’s room was categorized as frequent if cleaning occurred at least twice a week, occasional if cleaning occurred less than twice a week but more than once every 2 weeks, and rare if cleaning occurred less than once every 2 weeks [12]. Passive smoking refers to the exposure of a non-smoker to smoke from a lit cigarette or smoke exhaled by a smoker at least 1 day per week [13].

### Statistical analyses

A database was created using the EpiData software (version 3.1). A single-factor χ2 test was performed using the SPSS software (version 25.0). Variables that were significant in the single-factor χ2 test were selected for multivariable logistic regression analysis. A multivariable logistic regression model was used to analyse potential eczema risk factors. The factors with odds ratio > 1 as those that increased the risk of the eczema. *P* < 0.05 was considered statistically significant.

## Results

A total of 8153 preschool children were surveyed in this study (Table 1). The total number of people investigated was 10,000, yielding a response rate of 81.5%, 1847 children with incomplete responses to the questionnaire were excluded. The child cohort comprised 4235 (51.9%) boys and 3918 (48.1%) girls. The youngest and the oldest child were 2 years and 7.83 years old, and the average age was 5.27 ± 1.10 years. There were 7081 Han Chinese, accounting for 86.9% of the total, and 1072 belonging to other ethnic groups, accounting for 13.1% of the total.

**Table 1:** Prevalence of eczema among preschool children with different characteristics in Urumqi (*n* = 8153)

Characteristics	Number of people surveyed	Number of cases of recurrent rashes for 6 months in the past 12 months	Percentage (%)	*P*	Number of previous cases of recurrent rash for 6 months	Percentage (%)	*P*	Number of confirmed eczema cases	Percentage (%)	*P*
Sex										
Boys	4235	103	2.4	0.69	364	8.6	< 0.01	530	12.5	0.12
Girls	3918	90	2.3		271	6.9		447	11.4	
Nationality										
Han Chinese	7081	172	2.1	0.35	567	8.0	0.06	873	12.3	0.01
Other	1072	21	0.3		68	6.3		104	9.7	
Age (years)										
2–4	1033	31	3.0	0.34	83	8.0	0.93	112	10.8	0.48
4–6	4828	108	2.2		372	7.7		585	12.1	
6–8	2292	54	2.4		180	7.9		280	12.2	
Mode of birth										
Normal childbirth	4187	97	2.3	0.76	313	7.5	0.28	449	10.7	< 0.01
Cesarean delivery	3966	96	2.4		322	8.1		528	13.3	
Only child										
Yes	4569	112	2.5	0.57	374	8.2	0.13	605	13.2	< 0.01
No	3584	81	2.2		261	7.3		372	10.4	
Totals	8153	193	2.4		635	7.8		977	12.0	

The proportion of children who had recurrent rashes for 6 months in the past 12 months was 2.3%, with no significant differences between characteristics (all *P* > 0.05); 7.8% had previous history (prior to 12 months) of recurrent rash for 6 months, with higher incidence in boys than in girls (*P* < 0.05). The prevalence of participants with confirmed eczema was 12.0%, with 9.7% in Han Chinese and 12.3% in other ethnic groups. Moreover, there was higher incidence of eczema in children delivered through caesarean section than those having vaginal delivery delivery, and in only children than in children with siblings (all *P* < 0.05) (Table 1).

The survey revealed the following major environmental risk factors that can influence development of eczema in preschool children: purchase and use of new furniture and furnishings; mould or dampness in the living environment of mother before and during pregnancy and child’s first year; air conditioning; windows being open while sleeping; the presence of cockroaches, flies, or mosquitoes in the dwelling currently and during child’s first year; pets or flowering plants in the dwelling; passive smoking in the home environment currently, during the child’s first year and mother’s pregnancy; frequency of room cleaning; and family history of eczema (all *P* < 0.05) (Table 2).

**Table 2:** Single-factor analysis of eczema and indoor environmental variables (*n =* 8153)

Factors	Diagnosed with eczema		
	Number of cases	%	*P*
Housing area			
< 75 m2	239	12.1	0.91
≥ 75 m2	738	12.0	
Carpeting or mats in the home			
Yes	133	13.9	0.06
No	844	11.7	
Acquisition of new furniture in the dwelling of mother pre-pregnancy			
Yes	255	14.3	< 0.01
No	722	11.3	
Renovation of the dwelling of mother pre-pregnancy			
Yes	182	15.2	< 0.01
No	795	11.4	
Mold or dampness in the dwelling of mother pre-pregnancy			
Yes	176	20.1	< 0.01
No	801	11.0	
Acquisition of new furniture in the dwelling of mother during pregnancy			
Yes	135	15.5	< 0.01
No	842	11.6	
Renovation of the dwelling of mother during pregnancy			
Yes	100	17.0	< 0.01
No	877	11.6	
Mold or dampness in the dwelling of mother during pregnancy			
Yes	137	20.1	< 0.01
No	840	11.2	
Purchase of new furniture in the dwelling during the child first year			
Yes	117	16.0	< 0.01
No	860	11.6	
Renovation of the dwelling during the child first year			
Yes	85	17.5	< 0.01
No	892	11.6	
Mold or dampness in the dwelling during the child first year			
Yes	116	19.0	< 0.01
No	861	11.4	
Drying bedding			
Yes	883	12.1	0.37
No	94	11.0	
Use of air conditioning			
Yes	337	13.2	0.02
No	640	11.4	
Open window while sleeping			
Yes	893	12.4	< 0.01
No	84	8.9	
Cockroaches			
Yes	319	14.2	< 0.01
No	658	11.1	
Flies or mosquitoes			
Yes	778	13.4	< 0.01
No	199	8.5	
Pets in the child’s current dwelling			
Yes	203	17.5	< 0.01
No	774	11.1	
Flowering plants in the child’s current dwelling			
Yes	343	14.0	< 0.01
No	634	11.1	
Keeping pets in the dwelling during the child first year			
Yes	136	20.6	< 0.01
No	841	11.2	
Flowering plants in dwelling during the child first year			
Yes	274	14.7	< 0.01
No	703	11.2	
Passive smoking in the dwelling of child			
Yes	417	14.0	< 0.01
No	560	10.8	
Passive smoking in the dwelling during the child first year old			
Yes	359	14.6	< 0.01
No	618	10.9	
Passive smoking in the dwelling of mother during pregnancy			
Yes	315	15.3	< 0.01
No	662	10.9	
Frequency of room cleaning			
Regularly	809	11.6	0.02
Occasionally	156	14.5	
Very seldom	12	14.6	
Family history of eczema			
Yes	252	30.3	< 0.01
No	725	8.9	

The variables that were predicted to have a significant effect in the single-factor analysis together with whether the child had eczema as the dependent variable (0 = no; 1 = yes) were included in our multivariable logistic regression analysis. Eight variables that showed significant effects in the logistic regression model were associated with the onset of eczema. Among these, delivery method (OR = 1.18, 95%CI = 1.03–1.36), only child (OR = 1.36, 95%CI = 1.18–1.57), presence of mould or dampness in the mother’s home before pregnancy (OR = 1.53, 95%CI = 1.17–2.00), presence of flies or mosquitoes (OR = 1.31, 95%CI = 1.10–1.55), pets in the dwelling currently (OR = 1.23, 95%CI = 1.01–1.51), pets in the dwelling during child’s first year (OR = 1.45, 95%CI = 1.14–1.85), and family history of eczema (OR = 3.53, 95%CI = 2.98–4.19) were risk factors; ethnicity (not Han Chinese; OR = 0.77, 95%CI = 0.61–0.96) was considered as a protective factor against eczema (Table 3).

**Table 3:** Multifactorial logistic regression analysis of risk factors for eczema in preschool children in Urumqi (*n =* 8153)

Factors	Reference group	*P*	OR	95% CI
Ethnicity	Han ethnic group	0.02	0.77	0.61–0.96
Mode of birth	Normal childbirth	0.02	1.18	1.03–1.36
Only child	No	< 0.01	1.36	1.18–1.57
Mildew or dampness in the mother’s pre-pregnancy dwelling	No	< 0.01	1.53	1.17–2.00
Flies or mosquitoes	No	< 0.01	1.31	1.10–1.55
Pets in the child’s current residence	No	0.04	1.23	1.01–1.51
Keeping pets in the child’s residence when the child first year	No	< 0.01	1.45	1.14–1.85
Family history of eczema	No	< 0.01	3.53	2.98–4.19

*Abbreviations*: *OR* odds ratio, *CI* confidence interval

## Discussion

Eczema is a multivariable inflammatory skin disease, and the ISAAC study results suggested a median prevalence of eczema is 6.99% (0.95–22.5) in children aged 6–7 years in 132 centres [14]. The current study showed that 2.3% of preschool children aged 2–8 years in Urumqi, Xinjiang, reported symptom of recurrent rash in the past 12 months, 7.8% of children reported a previous history of recurrent rash for 6 months, and 12.0% of children reported having been diagnosed with eczema. Dampness in the living environment, the presence of flies or mosquitoes, and keeping pets indoors were risk factors for development of eczema. In a 2010 survey of preschool children in 10 Chinese cities, Zhang et al. [5] found a prevalence of eczema in 13.3% of children in Urumqi in the past 12 months, and Wang et al. [6] found a previous prevalence of eczema in 15% of children in 2011. These results along with the findings in other cities such as Wuhan [15] and Shenyang [4] indicate that Urumqi has a relatively low prevalence of eczema, which may be related to the environmental conditions in Urumqi.

Previous foreign studies found a significant association between father/mother having history of allergic disease and the development of eczema in infants [16, 17], suggesting the involvement of genetic factors in the development of eczema. Consistent with these findings, the present study substantiates family history of eczema as a risk factor for the development of eczema. The role of the filagrin gene *FLG* has been suggested in the development of eczema; *FLG* is the main gene aggregates intermediate keratin filaments, and patients with acute eczema have reduced *FLG* activity [18, 19]. Furthermore, animal studies conducted by Ge et al. [20] have suggested that acute eczema might be due to downregulated *FLG* expression and activation of protease-activated receptors and increased amount of transepidermal water loss in skin tissue, thereby disrupting skin barrier function and inducing eczema [20]. In addition, the *FLG* gene is prone to mutation, which can affect the integrity of the skin, thus reducing the skin’s barrier capacity against allergens. Invasion by foreign substances stimulates the antigen-presenting cells to activate Th2 cells, which then induces metamorphosis [21, 22].

The present survey found that ethnicity is a protective factor for eczema, with ethnic minorities having a lower prevalence of eczema than the Han Chinese, which is similar to the results of study by Zhao et al. [23], which demonstrated that the incidence of eczema was higher in the Han Chinese than in the Uyghur population in the spring, autumn, and winter. However, owing to the lack of comparative studies between the Han Chinese and other ethnic groups, the reasons for this are not yet known. In this study, we speculate that the reason for different incidence rates of eczema is not only related to the genetic differences between the ethnic minorities and Han population, but also most likely to be associated with the living habits of different ethnic groups. The people in Xinjiang have unique dietary habits, particularly the ethnic minorities, who prefer pasta, beef and mutton and eat less seafood. Studies have proposed that consumption of allergenic foods such as seafood by mothers during pregnancy and delivery is a key risk factor for the development of eczema in infants [24]. Other factors may include rest and activity patterns and family and marital relationships of ethnic minorities in Xinjiang, which differ greatly from those of the Han Chinese [22]. Therefore, the risk of eczema is lower in ethnic minorities than in the Han Chinese. Regarding delivery method, a number of studies have confirmed that caesarean section significantly affect development and maturation of the neonatal immune system likely owing to the imbalance of intestinal flora of these infants, leading to immune dysfunction and hyperactive immune responses that eventually cause occurrence of conditions such as allergies and autoimmunity [25, 26]. Consistent results were obtained in study by Azad et al. [27], the study demonstrated that the richness and diversity of the intestinal flora of infants delivered through caesarean section were low.

In addition, children also have a higher risk of eczema than only children, for which the cause is unknown. Although there is no literature to support this idea, we consider the possibility that non single children are more likely to have a fixed space for children’s activities but an increased number of active people, compared to single children, resulting in a rise in the density of the population in the room.

The presence of pets is associated with an increased levels of endotoxins and allergens in the environment. Several other studies have recognised household pets as a risk factor for the development of asthma and allergic disease [11, 28–30]. Although many studies have shown that exposure to such substances early in life is effective in reducing the risk of asthma and allergic diseases [31, 32]. This difference may be related to the climatic conditions; Xinjiang has a dry climate, which is suitable for the dispersal of endotoxins and allergens in the environment. Children are exposed to high levels of endotoxins and allergens by prolonged contact with pets, and are at an increased risk of eczema. Therefore, pets should be avoided in children’s homes. In addition, flies and mosquitoes were considered risk factors for the development of eczema in this study, but there is a lack of information on this aspect in China and abroad. In addition to eczema, other allergic diseases have been studied, however, the risk factors identified were the same, including the presence of pets causing onset of eczema and mosquito bites causing skin irritation. Since Xinjiang has a hot and dry climate, only few homes have damp problem. A humid environment is more suitable for mosquitoes to grow and breed along with the growth of dust mites and fungi in the house. According to Chen et al. [33], the second most common allergen for eczema is mould; the finding is consistent with the present study’s outcome identifying the presence of mould or dampness indoors as risk factors. Thus, good pest control and dehumidification can help reduce the incidence of eczema in children.

As this survey employed a cross-sectional study and was still in its initial stages, there may have been some bias in the data collection process. Additional cross-sectional studies have weak power for causal demonstration, and follow-up should also unfold population-based case-control studies or cohort studies that clearly influence eczema in preschool children. The study is one of the few investigations on eczema among preschool children that have been conducted in Urumqi City, this may be of some guidance for the prevention and treatment of eczema among children.

## Conclusion

Eczema has high risk among preschool children in Xinjiang, particularly the Han children. Delivery of children through caesarean section, presence of mould or moisture in the mother’s home before pregnancy, presence of flies or mosquitoes in the dwelling currently, pets kept in the child’s home currently, presence of pets during child’s first year, and family history of eczema, are suggested as risk factors for the development of eczema in children.

## Supplementary Information

**Additional file 1.**

## Data Availability

The datasets generated and/or analysed during the current study are not publicly available due the data belongs to the School of Public Health of Fudan University but are available from the corresponding author on reasonable request.

## Abbreviations

CI: Confidence interval
OR: Odds ratio
