# Supplementary material for: Prevalence and influencing risk factors of eczema among preschool children in Urumqi city: a cross-sectional survey
Source: BMC Pediatr. 2021 Aug 16;21:347. doi: 10.1186/s12887-021-02819-5 (PMC8365957; doi:10.1186/s12887-021-02819-5)
Supplement: Supplementary file 1 — Additional file 1. [file 12887_2021_2819_MOESM1_ESM.docx]

**Table S1. The processes of questionnaire survey.**

1. Six districts were randomly selected in Urumqi city according to their administrative divisions, which were the Xinshi District, the Shayibake District, the Tianshan District, the Shuimogou District, the Toutunhe District, and the Midong district.

2. In each of the 6 districts, 8-12 kindergartens were randomly selected, resulting in a total of 60 kindergartens surveyed.

3. The questionnaire was delivered to the Education Bureau of Urumqi city by the members of our subject group, to the strengths of the various head of each kindergarten by the Education Bureau, then to the teachers of all classes, and to the parents of the children by the teachers.

4. After the parents completed, the questionnaire was returned to the class director in place, the head of each kindergarten uniformly retracted, returned to the Education Bureau, and finally returned to this subject group by the Education Bureau.
